# Isolation and Identification of Subgroup J Avian Leukosis Virus Inducing Multiple Systemic Tumors in Parental Meat-Type Chickens

**DOI:** 10.3389/fvets.2020.614854

**Published:** 2021-01-27

**Authors:** Ning Cui, Xuezhi Cui, Qinghua Huang, Shaohua Yang, Shuai Su, Chuantian Xu, Jianhe Li, Wenfeng Li, Chao Li

**Affiliations:** ^1^Shandong Key Laboratory of Animal Disease Control and Breeding, Institute of Animal Science and Veterinary Medicine, Shandong Academy of Agricultural Sciences, Jinan, China; ^2^Shandong New Hope Liuhe Group Co., Ltd, Qingdao, China; ^3^Shandong Provincial Key Laboratory of Animal Biotechnology and Disease Control and Prevention, College of Veterinary Medicine, Shandong Agricultural University, Tai'an, China; ^4^Shandong Nongke Animal Husbandry Technology Co., Ltd, Jinan, China; ^5^Jinan Poultry Livestock Assistance Technology Co., Ltd, Jinan, China

**Keywords:** deletion, gp85 mutation, genome, multiple systemic tumors, avian leukosis virus

## Abstract

Avian leukosis virus (ALV) continues evolving to obtain new genomic characters to enhance its pathogenicity. In the present study, an ALV-J strain LH20180301 was isolated from broiler breeder chickens that reached the speak of paralyzation before 20-week-old. The necropsy chickens showed subcutaneous and muscular hemorrhage, and developed tumors in multiple organs including bone, liver, spleen, and kidney. The complete provirus was then cloned and sequenced to investigate the molecular characteristics and oncogenicity etiology of this virus associated with the outbreak of disease. The genomic structure of the reported ALV-J strain LH20180301 was highly conservative with other ALVs. Recombination events between the virus with endogenous virus were identified in the viral genome. Compared with the ALV-J original HPRS-103 strain, the major recombination sites of the viral genome with ev-1 were located in 5′ UTR-gag and 3′ UTR regions. Phylogenetic analysis of group specific antigen gp85 encoding protein showed that the LH20180301 branched with ALV-J prevalent in “yellow chickens” of local breeds in South China. Nine amino acids (N58, D60, K70, A71, K108, N112, N113, N121, R272) in the gp85 were highly conserved among ALV-J isolates before 2012, but various mutations were found in the late isolates including LH20180301. In addition, the LH20180301 strain also had the same deletion pattern of 3′ UTR with them. Therefore, LH20180301 might derive from the same ancestor with those viruses and may be the trend of ALV-J evolution in China. The defined new genomic characters in the gp85 and 3′ UTR region of ALV-J might provide the molecular basis for its enhanced oncogenicity.

## Introduction

Avian leukosis virus (ALV), the causative agent of avian leukosis (AL), is a member of the genus *Alpharetrovirus* of the family *Retroviridae* that associates with tumor formation, immunosuppression, and decreased fertility in birds (1). The members of subgroups A to E, J, and K mainly infect chickens in the field. The strain HPRS-103 was the first new subgroup J (ALV-J) isolate from a case of late-onset myelocytomas in broiler breeders and meat-type chickens in 1988 in the UK (2). ALV-J infection was first reported in China from broiler flocks in 1999 (3). The host range of ALV-J has expanded to layer-type birds, a number of native breeds of chickens, and even wild birds and wild ducks since 2007 (4, 5), leading to catastrophic economic loss in the poultry industry (6, 7). During the past decade, concerted efforts in eradication of the virus have successfully controlled the outbreak of AL in commercial breeding flocks in China. However, new outbreak of ALV-J associated with multiple systemic tumors since February 2018 bring huge loss to a number of chicken flocks in several provinces of China (8).

A number of factors have been reported to be associated with viral replication and tumorgenesis of ALVs. The gp85 glycoprotein is closely associated with antigenic affinity, tissue tropism and virulence, and it is the key factor for host infection and tumor formation (9, 10). Evidences have shown that amino acid mutations in the variable regions and the hypervariable regions of gp85 influence the specificity of virus (11). The 3′ UTR fragment was suspected to influence viral and host gene expression, which is critical in viral pathogenesis and tumor formation (12). The redundant transmembrane (rTM) might be associated with the viral virulence and evolution (13). The direct repeat 1 (DR1) region functions as a constitutive transport element and is important for replication of a virus (14). A unique 205-bp sequence covering the rTMs and DR1 regions enhances the replication capacity of ALV-J to increase its pathogenicity and induce incidence of hemangioma (15). The E element is closely related to the *src* gene and thus may play a role in oncogenesis (16). However, many of the oncogenic ALV-J showed various deletions in the E element, demonstrating that the region was not essential for oncogenesis (17, 18). The retroviral long terminal repeat (LTR) contains powerful transcription regulatory and enhancer elements (19–21). The special 11 bp deletion in U3 of LTR was vital important but not the determinant factor for ALV-J induced hemangioma (18, 22). In addition, due to the intrinsic nature of the *Retroviridae*, ALVs rapidly evolve by gene mutation and recombination, which promotes forming of new viral characteristics (23–25). Therefore, the mechanism of tumorigenesis is complicated and needs to be further studied.

In the present study, an ALV-J was isolated from broiler breeder chickens with a peak of paralyzation and multiple systemic tumors before 20-week-old. The complete provirus was cloned and sequenced to investigate the molecular characteristics and oncogenicity etiology of this virus associated with the outbreak of the disease.

## Materials and Methods

### Clinical Samples and Cells

Three flocks of broiler breeders from a commercial farm in Shandong province of China showed peak of paralyzation depression before 20-week-old in February 2018. Mortality for the flocks reached 20%. Three dead chickens were necropsied, and liver and kidney tissues were collected respectively for formalin fixation and storage at −80°C, respectively.

Chicken DF-1 cell was used for virus isolation and culturing (Kept in our laboratory). The cells were grown in Dulbecco's modified Eagle medium (DMEM, Invitrogen) supplemented with 10% fetal bovine serum (FBS, Gibco) and maintained in DMEM supplemented with 2% FBS at 37°C in a 5% CO_2_ incubator.

### Histopathological Assays

Liver and kidney tissues with typical lesion were collected and fixed in 10% formalin, and paraffin tissue sections were made. Sections were stained with hematoxylin and eosin (HE) for examination under a microscope (NIKON Eclipse ci) with a NIKON digital sight DS-FI2 imaging system.

### Virus Isolation and Identification

Virus isolation was performed on DF-1 cells as described previously (26). Briefly, clinical samples (including liver and kidney) were homogenized in PBS containing 100 U of penicillin and 100 μg of streptomycin per ml. Tissue homogenates were sterilized by a 0.22 μm filter and inoculated to DF-1 cells. After cultivation at 37°C in a 5% CO_2_ incubator for virus absorption for 2 h, the cells were maintained in DMEM supplemented with 2% FBS for 5 ~ 7 days. The cell supernatant was tested for ALV infection using the anti-p27 antibody-coated plates (IDEXX Inc., MA) following the manufacturer's instruction. Positive cells were collected for proviral DNA extraction using a Tissue DNA Extract Kit (TianGen). Env gene about 2200 nt in length was amplified and sequenced using the primer pair Env-F/R (Table 1) to define the subgroup of ALV.

**Table 1:** Primers used to amplify genes.

**Primers**	**Sequence (5^**′**^ → 3^**′**^)**	**Target gene (nt)**
Env-F	GAGGTGACTAAGAAAGATGAGGCGAGCC	2,200
Env-R	CCATCAACCCAGGTGCACACCAATG	
FC1-F	GCGTGTAGTGTTATGCAATACTC	2,827
FC1-R	ACTAATTGCGTTAGCGCTAC	
FC2-F	AGGAAGAGATTGTCTGCAGGGC	2,558
FC2-R	CCAAATAACCTTATCAGTGTCCCTG	
FC3-F	CTACTAGCCAAGGCAATGTATGC	2,483
FC3-R	TGAAGCCTTCTGCTTCATGCA	

An indirect immunofluorescence assays (IFA) was further performed to confirm the subgroup of the virus. The ALV-J specific monoclonal antibody JE9 at a dilution of 1:250 was used as primary antibody, and the FITC-conjugated goat anti-mouse antibody (Sigma, USA) at a dilution of 1:500 was used as secondary antibody. Uninfected DF-1 cells were used as negative control.

### Genome Cloning and Sequencing

The full-length of LH20180301 was amplified and sequenced in three segments. Primer pairs corresponding to the ALV-J prototype HPRS-103 were designed to amplify the viral genomic sequences (Table 1). PCR assays were performed using the proviral DNA as template for subsequent cloning. PCR products were cloned into pMD18-T vector (TaKaRa Co., Dalian), and three independent clones were sequenced by the Sangon Biotech (Shanghai, China) Co., Ltd.

### Phylogenetic and Recombination Analysis

Multiple sequence alignment with other ALV strains (Table 2) was carried out using DNASTAR software (DNASTAR, Madison, WI, USA) and the NCBI BLAST program (http://blast.ncbi.nlm.nih.gov/Blast.cgi). Major genes of the whole genome including gag, pol, gp37, gp85, and LTR were compared with other ALV strains. Phylogenetic analysis was performed using the neighbor-joining method with 1,000 bootstrap replicates (MEGA 6) (27). The 3′ UTR and important regulatory elements of U3 region of the isolate were analyzed using same programs. The possible recombination events were analyzed using the complete nucleotide sequence of LH20180301 by SimPlot software (V3.5.1) and mapped by Kimura (2-parameter) model. Reliability of recombination event was evaluated by 100 bootstrap and Parental Threshold > 70% was set as the cut-off value.

**Table 2:** ALV strains used for comparison of the sequence.

**Subgroup**	**Isolate (accession no.)**
A	RSV Schmidt-Ruppin A (L29199)
B	RSV Schmidt-Ruppin B (AF052428)
C	RSV-Prague C (J02342)
D	RSV Schmidt-Ruppin D (D10652)
E	ev-1 (AY013303), RAV-0 (XM73497)
J	HPRS-103 (Z46390), SD9901 (AY897220), SDC2000 (AY234052), NX0101 (DQ115805), NM20002-1 (HM235669), BJ0301 (AY897230), HA08 (HM235664), HAY013 (HM235665), JS-nt (HM235667), GDQY1201 (JX423792), GD13GZ (KU500030), GD1401J (KP317564), GD1407 (KU500034), GD14J2 (KU500032), SCAU-HN06 (KQ900844), SD07LK1 (FJ216405), NHH (HM235668), SCDY1 (HQ425636), SCAU11-H (KC149972), JL093-1 (JN624878), GD1109 (JX254901)
K	JS11C1(KF746200)

## Results

### Gross and Microscopic Lesions

**Figure 1:**
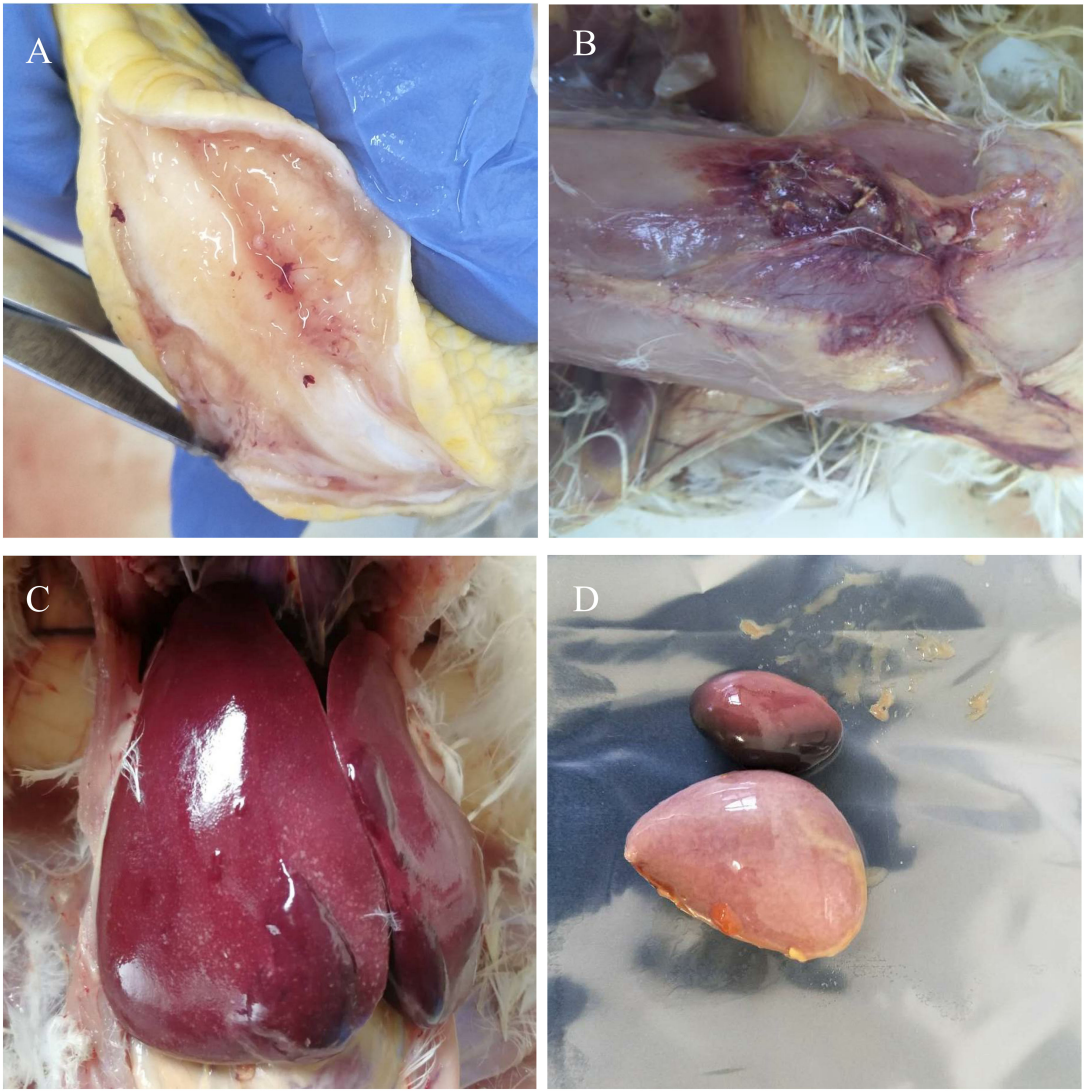
Gross lesions. Myeloid tumors in the bone **(A)** and liver **(C)**. Muscular hemorrhage **(B)** and spleen enlargement **(D)**.

**Figure 2:**
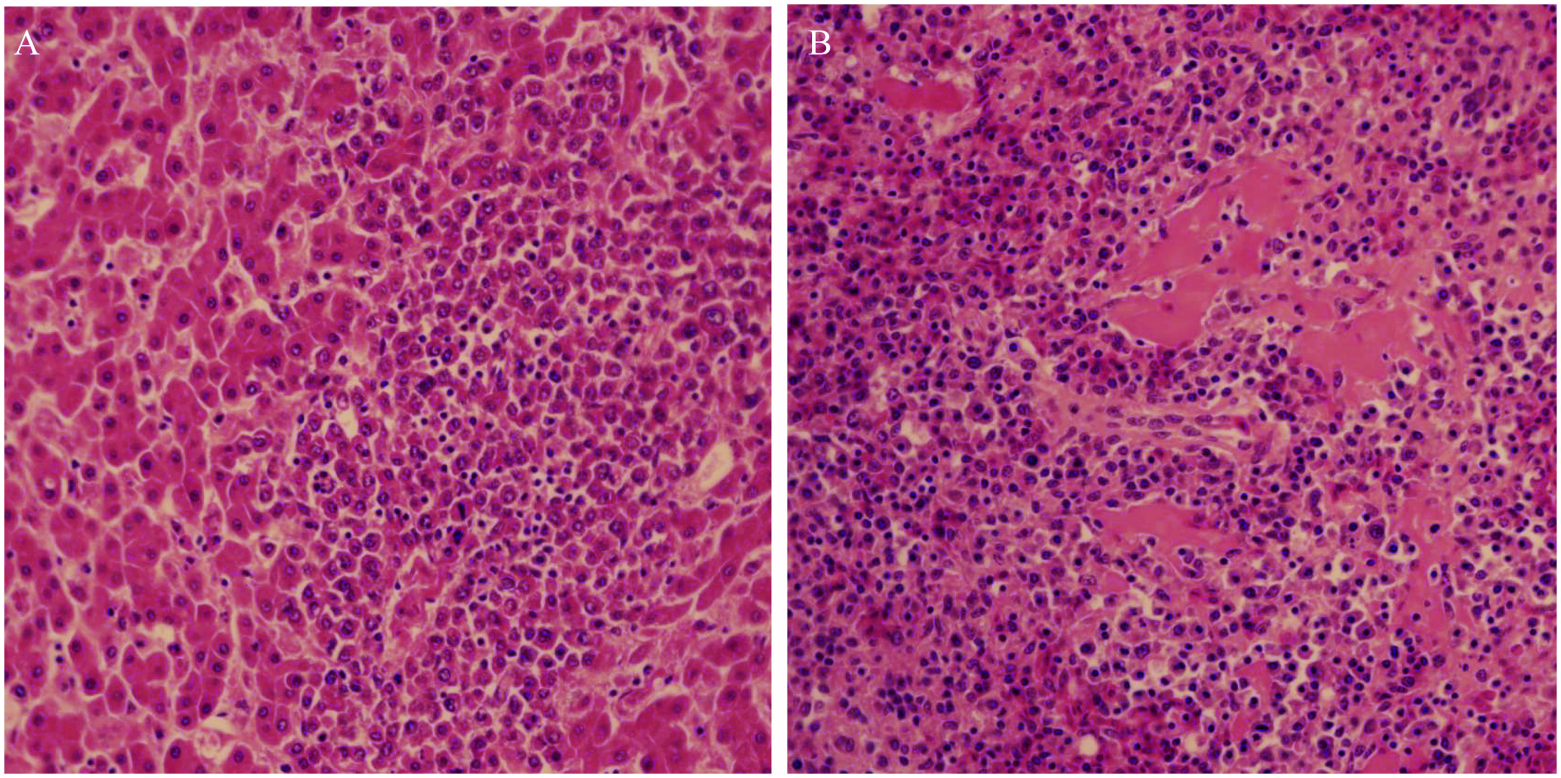
Histopathological lesions of the liver **(A)** and kidney **(B)** showing well-differentiated myeloid cells. Liver and kidney tissues with typical lesion were collected for pathology study.

Postmortem examination of the three birds showed subcutaneous and muscular hemorrhage, and different degree of widespread tumor-like infiltration in many organs, including the bone, liver, spleen, and kidney hemangioma (Figure 1). Tissue sections showed that massive well-differentiated myeloid cells with degenerated or necrotic hepatocytes were observed in the liver of all chickens (Figure 2A). There were tissue edema and tumor cell proliferation in the kidney tissue of all chickens (Figure 2B).

### Virus Detection and Isolation

**Figure 3:**
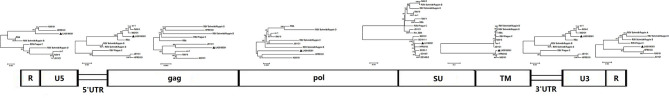
Segmental sequence comparison between the isolate LH20180301 and other ALVs. The genome sequences were divided by functional areas and multiple sequence alignment was carried out for each segmental sequence. Bottom box: genome structure of the isolate LH20180301. Phylogenetic tree: evolutionary relationships of each fragment inferred using the Neighbor-Joining method with 1000 bootstrap replicates (MEGA 6). Bar: substitutions per nucleotide position.

**Figure 4:**
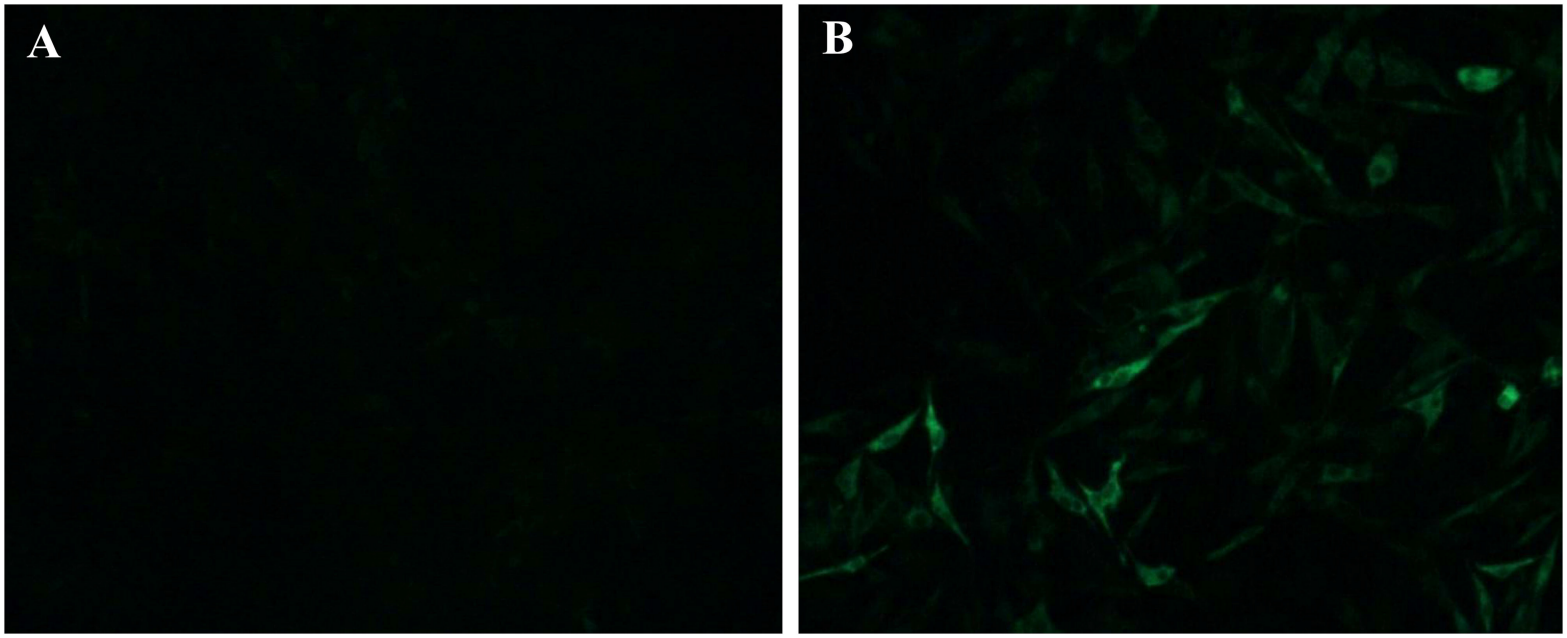
IFA identification of the isolate LH20180301. The ALV-J specific monoclonal antibody JE9 was used as primary antibody, and the FITC-conjugated goat anti-mouse antibody (Sigma, USA) was used as secondary antibody. **(A)** uninfected DF-1 cells; **(B)** DF-1 infected with LH20180301.

Clinical samples were homogenized and filtrated for inoculation to DF-1 cells. The infected DF-1 cells showed a positive result using ELISA assay with anti-p27 antibody-coated plates at 7 days post-inoculation, indicating the presence of exogenous ALV in the samples. Phylogenetic analysis of gp85 sequences of isolates indicated that the isolate clustered to ALV-J branch (Figure 3). An IFA assay with the anti-ALV-J monoclonal antibody JE9 showed that the virus infected DF-1 cells exhibited strong fluorescent light, further confirming that the isolate clustered to subtype J (Figure 4).

### Molecular Characterization of the Genome

**Figure 5:**
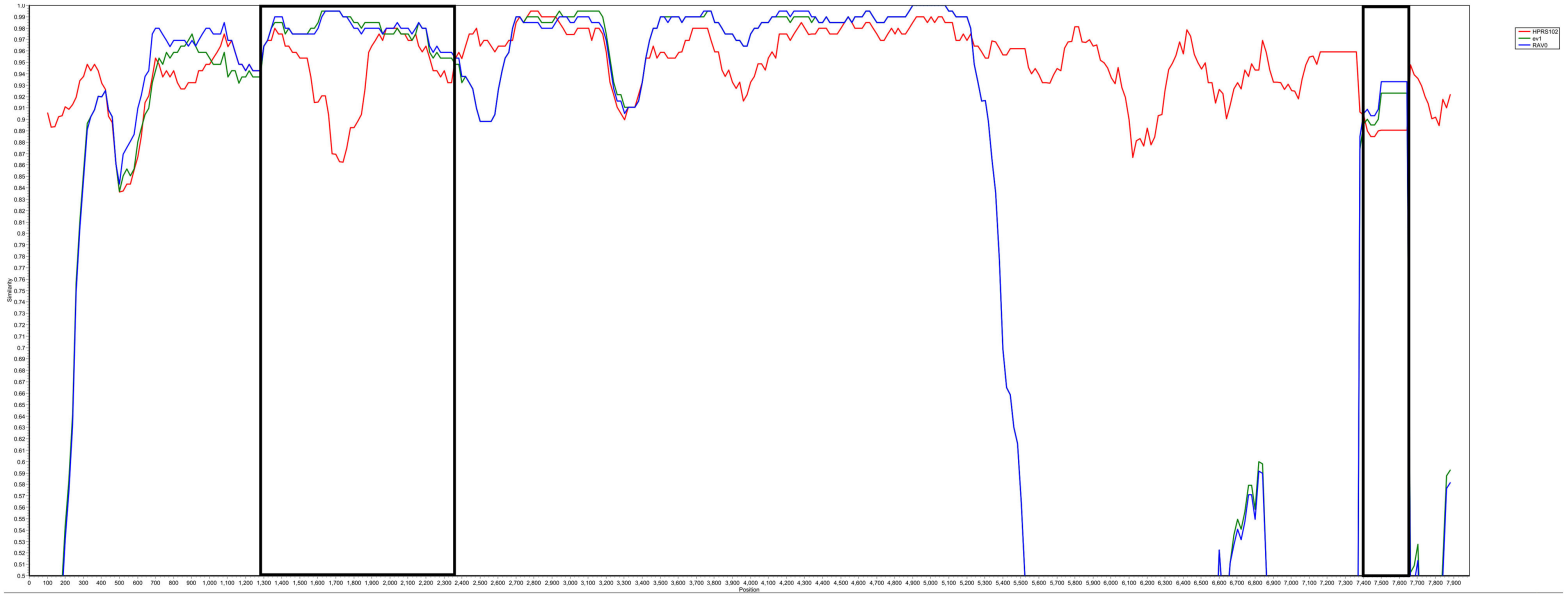
Analysis of LH20180301 recombination. Recombination events were analyzed by SimPlot software (V3.5.1) and mapped by Kimura (2-parameter) model. Reliability of recombination event was evaluated by 100 bootstrap and parental threshold > 70% was set as the cut-off value.

The complete genome sequences of LH20180301 was 7,471 nt in length and has been submitted to GenBank (Accession no, MK944404). A scheme of the genome structure of the strain LH20180301 and comparison with other ALVs were shown in Figure 3. Except for gp85 gene, all of the other genes including LTR region, 5′ UTRs, gag, pol, gp37, and 5′ UTRs gene of the LH20180301 are well-conserved with those of other ALVs with the homology above 96%. The gp85 gene showed the highest homology of 95.85% with an ALV-J isolate WA1112 from South China. Recombination events between the virus with ev-1 were identified in the viral genome. Compared with the ALV-J original HPRS-103 strain, the major recombination sites of the genome with ev-1 were located in 5′ UTR-gag and 3′ UTR regions (Figure 5). Further phylogenetic analysis confirmed that 5′ UTR-gag and 3′ UTR regions of LH20180301 clustered with ev-1 while the original HPRS-103 clustered with RSV Prague C and RSV Schmidt-Ruppin D. The remain part of the viral genome of LH20180301 and HPRS-103 were cluster to a branch (Figure 3).

### Molecular Characterization of gp85

**Figure 6:**
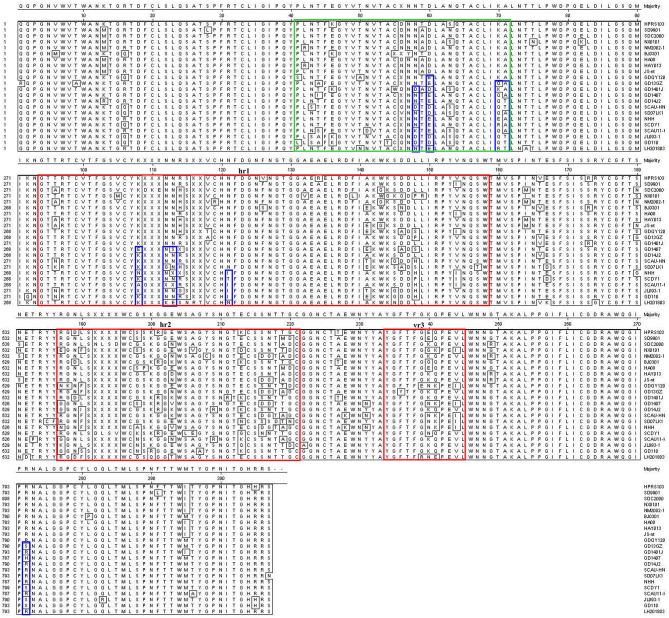
Comparison of amino acid sequences of gp85 of ALV-J isolates and the LH20180301 strain. Gp85 inferred was compared with other ALV strains. The red boxes indicate the hr1, hr2, and vr3 regions of gp85.

Most mutations in the gp85 glycoprotein of ALV-J were distributed in the hypervariable region 1 (hr1), hr2 and variable region 3 (vr3) regions (Figure 6). ALV-J isolates including LH20180301 evolved to form new characteristics in the gp85 glycoprotein in recent years. Nine amino acids (N58, D60, K70, A71, K108, N112, N113, N121, R272) in the gp85 gene were highly conserved before 2012, but various mutations were found in the late ALV-J isolates including LH20180301 strain. Four amino acids (K108, N112, N113, and N121) were located in the hr1 region and four amino acids (N58, D60, K70, and A71) were located in the peptide fragment from 41 to 72 amino acid site, which were reported for the first time. Phylogenetic analysis showed that the LH20180301 showed relatively high sequence homology to those viruses prevalent in “yellow chickens” of local breeds in South China and clustered to one branch (Figure 3).

### Molecular Characterization of 3′ UTR

**Figure 7:**
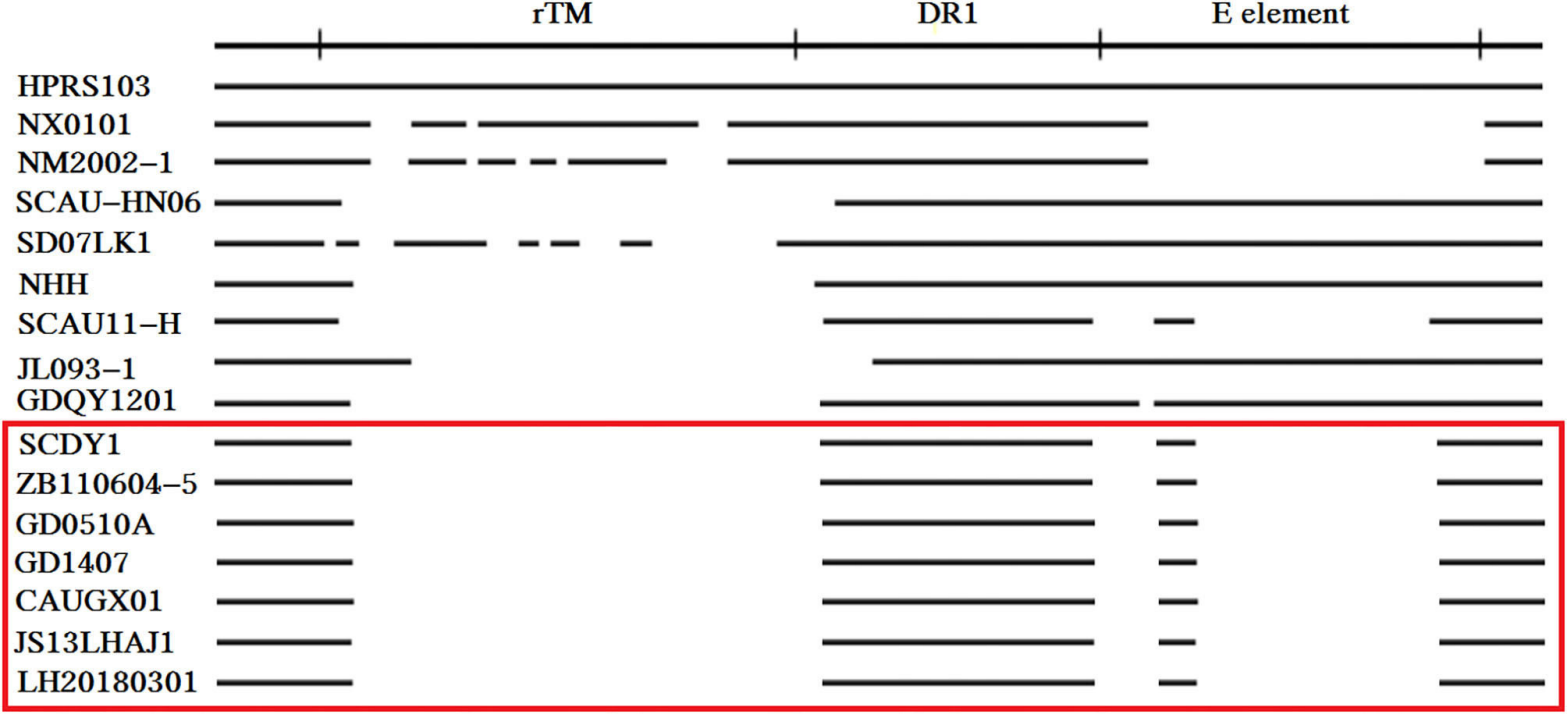
Comparison of the nucleotide deletions in the 3′ UTR of ALV-J isolates. The top line represents the elements in the genomic proviral DNA of HPRS-103. The deletions are indicated by empty spaces between the thick black lines.

Compared with the reported viruses, our isolate had the same deletion region of 3′ UTR with those viruses prevalent in “yellow chickens” of local breeds in southern China (GD0501A, GD1407, CAUGX01) and some isolates from Jiangsu and Sichuan. One isolate from gray partridge also had the same mutation. The 3'UTR gene has three elements, including the rTM region, DR1, and the E element. Further analysis showed that 210 nucleotides were deleted in the rTM region. The nucleotide sequence was conserved in the DR-1 region. Only 34 nucleotides were retained in the E element region (Figure 7).

### Molecular Characterization of U3 Region

**Figure 8:**
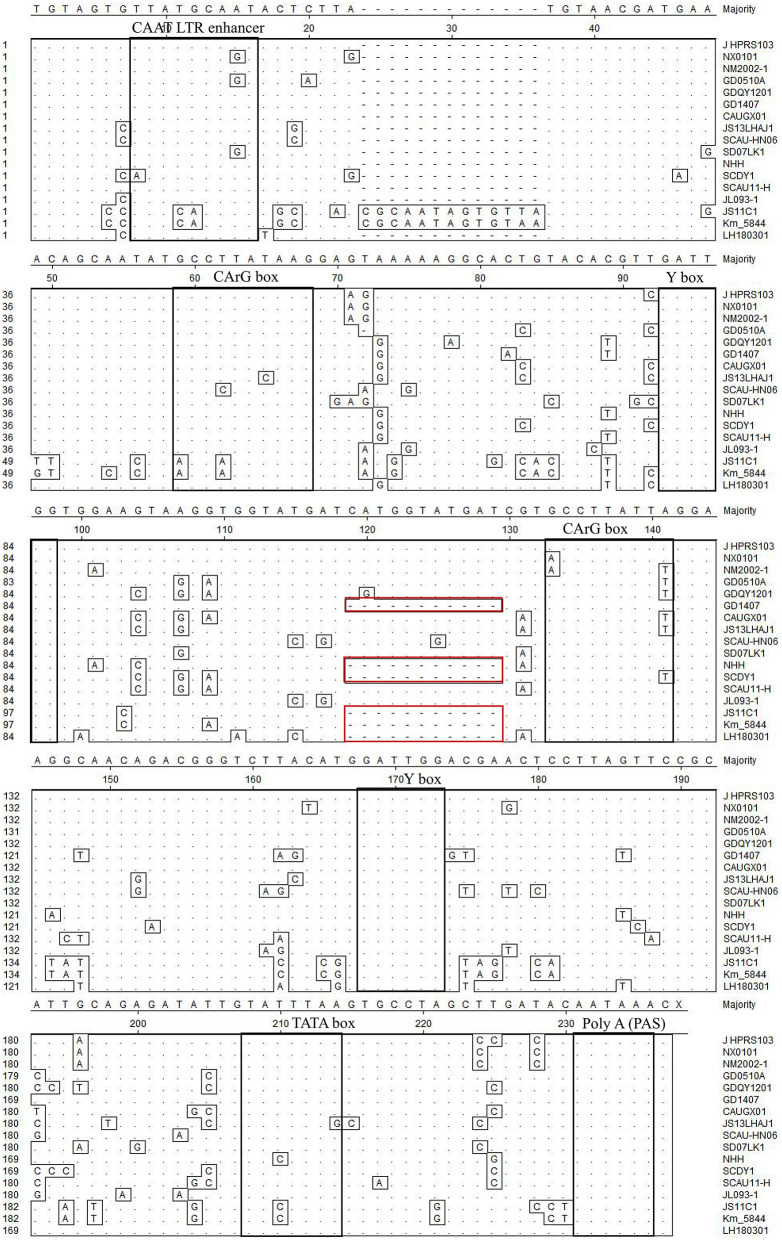
Comparison of nucleotides and important regulatory elements of U3 region among the LH20180301 and other ALVs. The dot (.) indicate identical residues, while the letters indicate base substitutions. The dashes(-) indicate gaps produced in the alignment. The locations of putative transcription regulatory elements are indicated in black boxes.

Transcriptional regulation elements of the LH20180301 are identified in the U3 region, including two CArG and Y boxes, one CAAT and TATA box (Figure 8). The putative transcription regulatory elements identified in the U3 region are relative conserved among ALV-J isolates. A total of 11 nucleotide deletions in the U3 region were observed in LH20180301 and other ALV-J isolates, including GD1407, NHH, and SCDY1. It is worth noting that the same deletion were also reported in some ALV-K isolates.

## Discussion

ALV-J brought huge economic losses in the poultry industry worldwide since its first isolation in the UK in 1988. Sporadic cases of ALV-J were reported in the recent years owning to the successfully eradication of the virus in commercial breeding flocks in China. The present study reported a new outbreak of ALV-J associated with multiple systemic tumors in several provinces of China in 2018. Coincidentally, a similar case of ALV-J threat with widespread tumor-like infiltration in many organs of the bird in the UK since its eradication was also reported in 2017 (28). Reemerge of ALVs around the world declares that the virus might evolve with new characters to enhance the virulence.

The genome structure of the reported ALV-J strain LH20180301 was highly conservative with other ALVs. Among the viral genes, gp85 showed relative lower homology with other viruses. The gp85 encoding protein is a ligand for receptor binding, which plays a vital role in viral entry and is the critical determinant of host ranges and tumor types (29). ALV-J is unique in its gp85 sequences as compared with other subgroups, which laid the molecular basis for the formation of myeloid tumors (10). Phylogenetic analysis showed that the LH20180301 branched with those viruses prevalent in “yellow chickens” of local breeds in South China and showed the highest homology of 95.85% with WA1112. Nine amino acids (N58, D60, K70, A71, K108, N112, N113, N121, R272) in the gp85 gene were highly conserved before 2012, but various mutations were found and considered as unique characteristics in the late ALV-J isolates including LH20180301 strain. Four amino acids were located in the hr1 region of a principal receptor interaction determinant (30). Four amino acids were located in the peptide fragment from 41 to 72, which were reported for the first time and appeared to be a new hypervariable region of highly oncogenic ALV-J since 2012. It is highly possible that the unique hypervariable region plays similar role as the hr1 that extended the tissue tropism of ALV-J in recent years (31). Recombination events between the virus with endogenous virus ev-1 were also identified in the viral genome. Compared with the ALV-J original HPRS-103 strain, the major recombination sites of the genome with ev-1 were located in 5′ UTR-gag and 3′ UTR regions. The above results suggested that the viruses evolved rapidly by recombination and continuous mutation to obtain new genomic characters, and the reported amino acid mutations and recombinations might have contributed to the molecular basis for the highly oncogenic characteristics of the virus.

The 3′ UTR of retrovirus evolved by nucleotide substitutions and deletions (15). The 3′ UTR in ALV-J mutated more frequently and exhibited different degrees of deletion since the first report of the prototype strain HPRS-103 (3). Our isolate had a 210 nucleotides deletion in the rTM region and there was only 34 nucleotides retained in the E element. The nucleotide sequence of DR-1 region was highly conserved. It was believed that the DR-1, together with the conserved two GArG boxes and two Y boxes, function as a constitutive transport element and guarantee the viral replication ability in the host (14). The deletion pattern of 3′ UTR was the same as those viruses prevalent in “yellow chickens” of local breeds in southern China (GD0501A, GD1407, CAUGX01) and some isolates from Jiangsu and Sichuan (18, 22, 32). Those isolates mostly showed earlier onset of disease and more severe pathogenesis in multiple organs (5, 18). Tumors occurred as early as 20-week-old in parental meat-type chickens in the field cases (5). It is extremely possible that the unique deletions in the 3′ UTR were related to the disease progress, and the precise role of which remains to be investigated in the future.

The ALVs lacking oncogenes induce neoplasms in chickens by integrating LTR fragment into or near the proto-oncogene or tumor suppressor genes of the host and perturbing their expression (33). A total of 11 nucleotide deletions in the U3 region of LTR were observed in LH20180301 and other ALV-J isolates, including GD1407, NHH, and SCDY1. The 11 nucleotide deletions usually observed in layer isolates associated with hemangioma (22). However, it is worth noting that the same deletion were also reported in some ALV-K isolates, supporting that such genome character is not unique to virulent ALV-J and is not the determinant factor for ALV-J induced hemangioma (18). We consider that the virus might evolve due to selection pressure regardless of virus subtypes. Combined with the phylogenetic analysis and mutation of gp85, and the same deletion pattern of 3′ UTR with ALV-J prevalent in “yellow chickens” of local breeds in South China, these viruses might derive from the same ancestor and spread around many provinces of China. Thus, ALV-J isolates may have been persistently circulating at a low level in the chicken flocks and evolved with new characters to reemerge as high pathogenic ALV-J strain associated with multiple systemic tumors. It further highlights the importance of continued surveillance and genomic analysis of ALVs.

## Conclusion

An ALV-J strain LH20180301 isolated from broiler breeder chickens with peak of paralyzation and multiple systemic tumors before 20-week-old was reported. Full genome analysis revealed that the virus evolved by recombination and continuous mutation. Segment alignment of viral genome showed that the gp85 glycoprotein of LH20180301 branched with ALV-J isolates prevalent in “yellow chickens” of local breeds in South China and had the same deletion pattern of 3′ UTR with them. New amino acids mutation characters in the gp85 were first found in the late ALV-J isolates including LH20180301. Our results supported that LH20180301 might derived from the same ancestor with those viruses. The defined new genomic characters in the gp85 and 3′ UTR region might provide the molecular basis for its enhanced oncogenicity.

## Data Availability Statement

The datasets presented in this study can be found in online repositories. The names of the repository/repositories and accession number(s) can be found at: https://www.ncbi.nlm.nih.gov/genbank/, MK944404.

## Author Contributions

All authors listed have made a substantial, direct and intellectual contribution to the work, and approved it for publication.

## Conflict of Interest

XC was employed by company New Hope Liuhe Group Co., Ltd. JL and CL were employed by company Shandong Nongke Animal Husbandry Technology Co., Ltd. WL was employed by Jinan Poultry Livestock Assistance Technology Co., Ltd. The remaining authors declare that the research was conducted in the absence of any commercial or financial relationships that could be construed as a potential conflict of interest.
